# The Brain-Uterus Connection: Brain Derived Neurotrophic Factor (BDNF) and Its Receptor (Ntrk2) Are Conserved in the Mammalian Uterus

**DOI:** 10.1371/journal.pone.0094036

**Published:** 2014-04-08

**Authors:** Jocelyn M. Wessels, Liang Wu, Nicholas A. Leyland, Hongmei Wang, Warren G. Foster

**Affiliations:** 1 Department of Obstetrics and Gynecology, McMaster University, Hamilton, Ontario, Canada; 2 State Key Laboratory of Reproductive Biology, Institute of Zoology, Chinese Academy of Sciences, Beijing, China; University of Tennessee Health Science Center, United States of America

## Abstract

The neurotrophins are neuropeptides that are potent regulators of neurite growth and survival. Although mainly studied in the brain and nervous system, recent reports have shown that neurotrophins are expressed in multiple target tissues and cell types throughout the body. Additionally, dysregulation of neurotrophins has been linked to several disease conditions including Alzheimer's, Parkinson's, Huntington's, psychiatric disorders, and cancer. Brain derived neurotrophic factor (BDNF) is a member of the neurotrophin family that elicits its actions through the neurotrophic tyrosine receptor kinase type 2 (Ntrk2). Together BDNF and Ntrk2 are capable of activating the adhesion, angiogenesis, apoptosis, and proliferation pathways. These pathways are prominently involved in reproductive physiology, yet a cross-species examination of BDNF and Ntrk2 expression in the mammalian uterus is lacking. Herein we demonstrated the conserved nature of BDNF and Ntrk2 across several mammalian species by mRNA and protein sequence alignment, isolated *BDNF* and *Ntrk2* transcripts in the uterus by Real-Time PCR, localized both proteins to the glandular and luminal epithelium, vascular smooth muscle, and myometrium of the uterus, determined that the major isoforms expressed in the human endometrium were pro-BDNF, and truncated Ntrk2, and finally demonstrated antibody specificity. Our findings suggest that BDNF and Ntrk2 are transcribed, translated, and conserved across mammalian species including human, mouse, rat, pig, horse, and the bat.

## Introduction

Brain derived neurotrophic factor (BDNF) is one member of the neurotrophin family of secreted growth factors which also comprises nerve growth factor (NGF), neurotrophin-3 (Ntf3), and neurotrophin-4/5 (Ntf5). The neurotrophins are classically known for their participation in the development, growth, function, and survival of neurons in both the central and peripheral nervous system [1]. They induce a myriad of actions by signalling through the neurotrophic tyrosine receptor kinase family (Ntrk1 – formerly TrkA, Ntrk2 – formerly TrkB, Ntrk3 – formerly TrkC, and NGFR – formerly p75NTR). BDNF binds with a high affinity to Ntrk2, which has at least three isoforms, a full length transmembrane receptor, and two truncated receptors. Mainly studied in the nervous system, the interaction between BDNF and the full length Ntrk2 receptor has also been shown to activate adhesion, angiogenesis, apoptosis, and proliferation pathways via the ras-mitogen-activated protein kinase (MAPK), phosphatidylinositol 3-kinase (PI3K), and the phospholipase Cγ1-Ca^2+^ pathway [1]–[3]. In addition to participating in many physiological processes, the neurotrophins have been linked to numerous pathologies (Alzheimer's, Parkinson's, Huntington's, cancer) and psychiatric disorders (bipolar, schizophrenia, depression) [1], [4], [5].

Although abundant in the nervous system, BDNF and Ntrk2 are expressed in other cell types and tissues, and *BDNF* mRNA is found in the majority of the human body organs [6]. In humans, mature BDNF is sequestered in platelets [7] and released upon their degranulation. As such, BDNF has access to all tissues and organs. Motile cells including activated T cells, B cells, and monocytes have been shown to express BDNF *in vitro* [8], [9], as have eosinophils [10], dendritic cells [11], and endothelial cells [12]. In mice, the visceral epithelium [13], and airway epithelium are significant sources of BDNF [14]. As for Ntrk2, a comprehensive analysis of Ntrk2 immunoreactivity was assessed and it was found to be expressed mainly in glandular cells of the salivary gland, small intestine, colon, endocrine pancreas, bone marrow hematopoietic cells, monocytes/macrophages of the lymph nodes and spleen, and in the epidermis [15]. Previous studies have shown that neurotrophins in the brain are regulated by neuronal activity (Ca++ influx induced transcription) [16], and steroid hormones [17]–[20], and that tissue-specific expression is driven by multiple promoters [21].

Although the interaction between the BDNF-Ntrk2 ligand-receptor pair has been shown to activate the adhesion, angiogenesis, apoptosis, and proliferation pathways in other body systems, very few studies have addressed their physiological role in reproduction. While BDNF and Ntrk2 expression has been demonstrated in some reproductive tissues including the ovary [22], [23], and placenta [24], their uterine expression under physiological conditions has been questionable. BDNF expression was demonstrated by immunohistochemistry in the mouse [25], and human uterus [26], [27] and by *in situ* hybridization in the mouse [13], and rat [18] uterus. While Ntrk2 could not be detected in the mouse [13] and human uterus [15], others have been successful [28], [29]. To date only one study has looked for the presence of both ligand and receptor simultaneously, in the murine uterus [13]. Moreover, the uterine expression of BDNF and Ntrk2 has not been examined in species other than the mouse, rat, and human.

Herein we present a comprehensive overview of the conserved nature of BDNF and Ntrk2 expression in the uterus of several mammalian species including human, mice, rats, pigs, horses, and bats.

## Materials and Methods

### GenBank Accession Numbers

Human BDNF (KC855559), Mouse BDNF (KC855560), Rat BDNF (KC855561), Pig BDNF (KC855563), Horse BDNF (KC855562), Human Ntrk2 (KC855566), Mouse Ntrk2 (KC855567), Rat Ntrk2 (KC855568), Horse Ntrk2 (KC855569).

### Cross-Species mRNA and Protein Sequence Alignment

mRNA and protein sequences were obtained for coding regions of the Ntrk2 and BDNF genes from sequences available on NCBI's Nucleotide (www.ncbi.nlm.nih.gov/nuccore). mRNA was aligned across species using mVISTA (http://genome.lbl.gov/vista/mvista/submit.shtml), and phylogenetic trees were constructed [21]–[33]. NCBI's Blastn and Blastp were used to compare nucleotide and protein identities and gaps between species.

### Animal and Human Samples

#### Ethics statement

All animal procedures followed research protocols approved by the Animal Research Ethics Board at McMaster University, the University of Guelph Animal Care Committee, and the Ethical Committee, State Key Laboratory of Reproductive Biology, Institute of Zoology, Chinese Academy of Sciences. Collection of human endometrial tissue samples was approved by the McMaster University and Hamilton Health Sciences Research Ethics Board (REB #10-326-T) and written informed consent was provided by study participants.

#### Mice

C57/Bl6 mouse uterine horns (n = 31) were collected from non-pregnant females aged 8–12 weeks, post-euthanasia and were promptly placed on ice. One uterine horn was stored at −80°C until required. The other was placed in 10% formalin, processed, and embedded in paraffin wax for immunohistochemistry.

#### Rats

The uterine horns of non-pregnant female Wistar rats (n = 11) were graciously provided by Dr. Alison Holloway. Uterine horns were collected at euthanasia and immediately placed on ice. One uterine horn was stored at −80°C until required. The other was placed in 10% formalin, processed, and embedded in paraffin wax for immunohistochemistry.

#### Humans

Human uterine samples (n = 8) were collected by the Pathology Department at McMaster University Medical Centre (Hamilton, ON, Canada) from patients undergoing a hysterectomy. Samples were immediately transported to the lab, and bisected with one half being frozen for RNA/protein applications, and the other half placed in 10% formalin, processed, and embedded in paraffin wax for immunohistochemistry.

#### Pigs

Non-pregnant porcine uterus (n = 3) was provided by Dr. Chandra Tayade. Samples were collected at euthanasia, placed on ice, and one half was frozen at −80°C until required. The other was placed in 4% paraformaldehyde, processed, and embedded in paraffin wax for immunohistochemistry.

#### Horses

Archived uterine punch biopsies previously obtained from five pregnant mares at gestation day 15 (n = 5) were provided by Dr. Keith Betteridge. RNA from three biopsies was available and two biopsies had been processed for immunohistochemistry. Non-pregnant uterine tissue was not available for study.

#### Bats

All procedures were carried out in accordance with the Policy on the Care and Use of Animals, approved by the Ethical Committee, State Key Laboratory of Reproductive Biology, Institute of Zoology, Chinese Academy of Sciences. Collection of the uterine horns of fulvous fruit bats was detailed previously [34]. In brief, the bats were trapped alive on Day 21 (n = 6; the day when menstrual bleeding was observed was designated as Day 1). The uterine horns were collected under anesthesia, fixed in 4% paraformaldehyde solution, dehydrated with graded ethanol solution, and then processed for paraffin embedding.

### RNA and Protein Extraction

Total RNA was extracted from all mouse, rat, and human endometrial samples using the RNA/Protein Plus kit (Norgen Biotek, Mississauga, ON, Canada). The protocol was modified slightly from the manufacturer's directions. Briefly, approximately 25 mg of frozen uterus was minced with a scalpel, placed in 300 μl of lysis reagent from the kit, and disrupted on ice using a sonicator (Fisher Scientific, Ottawa, ON, Canada) for roughly 5 seconds. Samples were centrifuged at 4°C at 13000 rpm for 2 minutes. Genomic DNA was removed using a column separator from the RNA/Protein Plus kit, and the remainder of the procedure was performed according to the protocol provided. RNA concentration and quality were assessed by spectrometry (Beckman Coulter, Mississauga, ON, Canada). RNA was extracted from horse and pig endometrium using the RNeasy kit (Qiagen, Mississauga, ON, Canada) according to the manufacturer's directions. RNA concentration and purity was measured using the GeneQuant pro RNA/DNA calculator (Biochrom Ltd., Cambridge, UK).

Protein extraction from human endometrium (n = 8) and mouse brain as a positive control was performed in 200 μl of RIPA buffer. The tissue was disrupted on ice using a sonicator three times, for 5 seconds. Samples were centrifuged, and the supernatant collected. Protein concentration was measured on a microplate reader at 595 nm using the Bio-Rad protein assay based on the Bradford method (Bio-Rad, Mississauga, ON, Canada).

### Real-Time PCR

RNA from mouse, rat, human, pig, and horse was reverse transcribed using the iScript cDNA synthesis kit (Bio-Rad), according to kit protocol. PCR primers were designed using human GenBank sequences for BDNF mRNA (NM_001143809.1) and Ntrk2 mRNA (NM_006180.3). Primers were designed against a 300 bp span within the coding region of the gene, and whenever possible were designed to span an intron. Primer3 software (http://frodo.wi.mit.edu/primer3/) was used for primer design and primers were tested for hairpins, self-dimers, and hetero-dimers using OligoAnalyzer 3.1 (http://www.idtdna.com/analyzer/applications/oligoanalyzer/). Primer sequences for *BDNF* were (Forward: GAGCTGAGCGTGTGTGACAG, Reverse: CTTATGAATCGCCAGCCAAT), and for *Ntrk2* (Forward: CAATTGTGGTTTGCCATCTG, Reverse: TGCAAAATGCACAGTGAGGT). Primers were ordered from Mobix Laboratory (McMaster University, Hamilton, ON, Canada), and diluted to a working concentration of 10 pmol/μl with DNase/RNase free water.

cDNA for 3 animals per group was pooled and used to isolate *BDNF* and *Ntrk2* transcripts. Real-Time PCR was performed in triplicate in a 10 μl reaction volume (2 μl pooled cDNA, 5 μl SYBR Green Master Mix (Qiagen), 1 μl forward primer, 1 μl reverse primer, and 1 μl RNase/DNase free water) using the capillary-based LightCycler (Roche Diagnostics, Laval, QC, Canada). The program was denaturation: 95°C for 15 min; amplification: 55 cycles: 95°C for 10 s, 56°C for 5 s, 72°C for 20 s; melting curve: 70–95°C at a rate of 0.1°C per second. Amplification and melt curves were analyzed for each species using the LightCycler software (Roche Diagnostics). PCR products were collected, and sent for sequencing (Laboratory Services, University of Guelph). Each sequence was searched under the BLASTN analysis on the National Center for Biotechnology Information website. Sequences were submitted to NCBI GenBank (accession numbers and PCR product melting temperatures are listed in Table 1).

**Table 1:** GenBank accession numbers and Real-Time PCR melting peak temperatures.

Species and Gene	GenBank Accession	Melting Peak (°C)
Human *BDNF*	KC855559	82
Mouse *BDNF*	KC855560	82
Rat *BDNF*	KC855561	82
Pig *BDNF*	KC855563	84
Horse *BDNF*	KC855562	84
Human *Ntrk2*	KC855566	79
Mouse *Ntrk2*	KC855567	82
Rat *Ntrk2*	KC855568	83
Horse *Ntrk2*	KC855569	80

### Assessing Antibody Specificity

#### Antibody Pre-absorption

Mouse brain sections were cut at a thickness of 4 μm, and incubated with 1) anti-BDNF or anti-Ntrk2 1∶200 (Abcam, Cambridge, MA, USA) (positive control), 2) anti-BDNF or anti-Ntrk2 pre-incubated with an excess of human recombinant protein (BDNF Abcam ab9794 and Ntrk2 Abcam ab56652) at a 5∶1 ratio with the antibody, or 3) normal goat serum *in lieu* of primary antibody. BDNF sections were counterstained with propridium iodide, and visualized using a chicken anti-rabbit Alexa Fluor 488 secondary (Life Technologies, Burlington, ON, Canada). Fluorescence was captured using the Photometrics CoolSnap HQ camera (Roper Scientific, Sarasota, FL, USA) and identical exposure times between positive, preabsorbed, and negative sections. Ntrk2 sections were stained using the ABC kit (Vector Labs, Burlington, ON, Canada) and DAB as a chromogen, and images captured with an Infinity camera (Lumenera Corp., Ottawa, ON, Canada) under 200X magnification on an Olympus IX81 microscope (Olympus, Richmond Hill, ON, Canada).

#### Human Recombinant Protein Western Blot

Antibody specificity was also assessed by Western Blot (as below) using the same recombinant human BDNF and truncated Ntrk2 proteins as above (Abcam) in a 2X serial dilution.

### Immunohistochemistry

Paraffin sections were cut at a thickness of 4 μm for mice (n = 31), rats (n = 11), humans (n = 10), pigs (n = 3), and horses (n = 2). Sections were separately stained for BDNF and Ntrk2 using a 1∶200 dilution of rabbit anti-BDNF (Abcam) or rabbit anti-Ntrk2 (Abcam), as above. Negative sections were incubated with normal goat serum *in lieu* of primary antibody. Images were captured by an Infinity camera (Lumenera Corp.) under 200X magnification on an Olympus IX81 microscope. Bat sections (n = 6) were stained at the State Key Laboratory of Reproductive Biology, Institute of Zoology, Chinese Academy of Sciences, in Beijing, China using anti-BDNF (Santa Cruz Biotechnology Inc., Dallas, TX, USA) and anti-Ntrk2 (Santa Cruz) antibodies as above.

### Western Blot

Extracted protein (60 μg) from human endometrium was run on a 4–20% gradient gel (Thermo-Scientific) at 150 V for 50 minutes. Protein was transferred to PVDF membrane (VWR International, Mississauga, ON, Canada) at 40 V for 90 minutes. Blots were blocked for 1 hour at room temperature with 5% skim milk/TBS-T, and subsequently probed with 1∶1000 rabbit anti-BDNF (Abcam) or 1∶1000 rabbit anti-Ntrk2 (Abcam), overnight at 4°C. Anti-Rabbit-ECL secondary (GE, Mississauga, ON, Canada) at a concentration of 1∶5000 was applied for 1 hour at room temperature, blots were briefly washed in TBS-T and TBS, then incubated with ECL substrate (Thermo-Scientific) for 5 minutes. Exposures were performed using x-ray film (Thermo-Scientific), and the exposure times were 60, and 45 minutes for BDNF and Ntrk2 respectively.

## Results

### Cross-Species mRNA and Protein Sequence Homology

**Figure 1:**
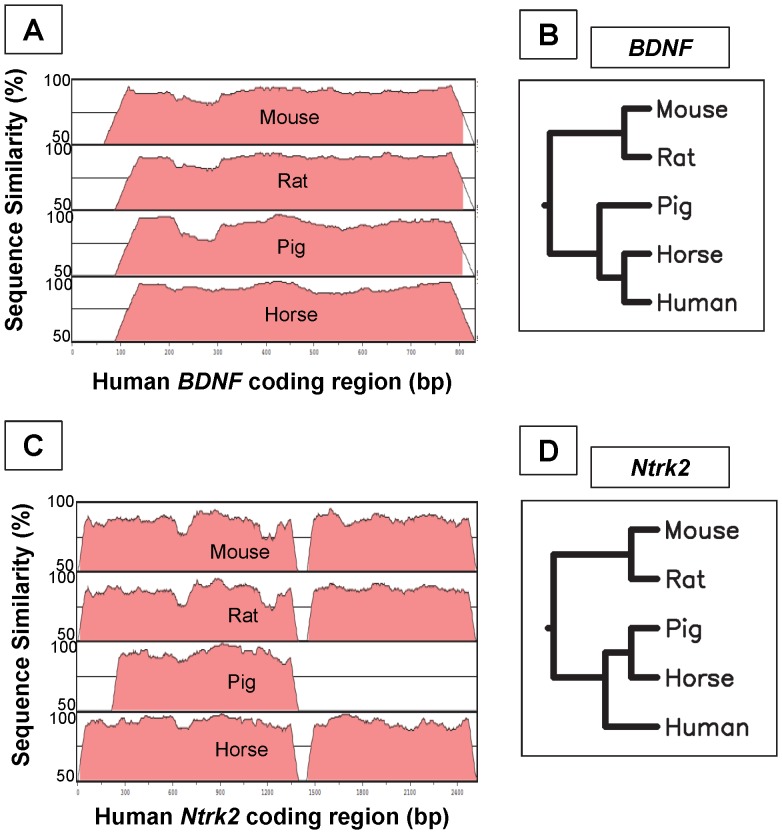
Sequence Homology between Species. Coding regions for *BDNF* (A) and *Ntrk2* (C) were aligned between human, mouse, rat, pig, and horse using mVISTA to show inter-species similarities. Results are displayed as percent conservation between all species as compared to the human sequence. Phylogenetic trees were created for *BDNF* (B) and *Ntrk2* (D) to visually illustrate which species were most closely related. bp: base pairs.

When the coding regions of the BDNF and Ntrk2 genes were compared, they were very homologous between the species examined (human, mouse, rat, pig, horse). The mRNA for both genes had less homology between species as compared to the protein. BDNF mRNA ranged from 90–98% (Table 2), and protein from 95–99% (Table 3). Ntrk2 mRNA ranged from 84–94% (Table 4), and protein from 87–99% (Table 5). The mRNA coding region from mouse, rat, pig, and horse for both *BDNF* (Figure 1A) and *Ntrk2* (Figure 1C) was aligned against the human sequence and are displayed as percent conservation between all of the aligned species as compared to the human sequence. Phylogenetic trees were created for each mRNA to determine which species were most closely related (Figure 1B, D).

**Table 2:** Comparison of the coding region of *BDNF* mRNA across species.

Species	Comparison Species	Base Pair Identities (*BDNF* mRNA)	Gaps
Human	Mouse	712/775 (92%)	0%
	Rat	687/750 (92%)	0%
	Horse	690/744 (93%)	0%
	Pig	691/759 (91%)	1%
Mouse	Rat	735/750 (98%)	0%
	Horse	676/750 (90%)	0%
	Pig	681/759 (90%)	1%
Rat	Horse	682/750 (91%)	0%
	Pig	686/759 (90%)	1%
Horse	Pig	692/759 (91%)	1%

**Table 3:** Comparison of the coding region of BDNF across species.

Species	Comparison Species	Amino Acid Identities (BDNF Protein)	Gaps
Human	Mouse	248/256 (97%)	0%
	Rat	241/249 (97%)	0%
	Horse	240/247 (97%)	0%
	Pig	244/252 (97%)	1%
Mouse	Rat	247/249 (99%)	0%
	Horse	237/249 (95%)	0%
	Pig	241/252 (96%)	1%
Rat	Horse	239/249 (96%)	0%
	Pig	241/252 (96%)	1%
Horse	Pig	240/252 (95%)	1%

**Table 4:** Comparison of the coding region of *Ntrk2* mRNA across species.

Species	Comparison Species	Base Pair Identities (*Ntrk2* mRNA)	Gaps
Human	Mouse	1211/1397 (87%)	0%
	Rat	2160/2517 (86%)	2%
	Horse	2302/2517 (91%)	2%
	Pig	1090/1180 (92%)	0%
Mouse	Rat	2325/2466 (94%)	0%
	Horse	2155/2471 (87%)	0%
	Pig	1003/1182 (85%)	1%
Rat	Horse	2149/2472 (87%)	0%
	Pig	991/1182 (84%)	1%
Horse	Pig	1105/1184 (93%)	0%

**Table 5:** Comparison of the coding region of Ntrk2 across species.

Species	Comparison Species	Amino Acid Identities (Ntrk2 Protein)	Gaps
Human	Mouse	772/838 (92%)	2%
	Rat	769/838 (92%)	2%
	Horse	800/838 (95%)	2%
	Pig	376/394 (95%)	0%
Mouse	Rat	809/821 (99%)	0%
	Horse	767/822 (93%)	0%
	Pig	348/393 (89%)	0%
Rat	Horse	768/822 (93%)	0%
	Pig	348/393 (89%)	0%
Horse	Pig	393/452 (87%)	4%

### *BDNF* and *Ntrk2* Transcripts in the Uterus

**Figure 2:**
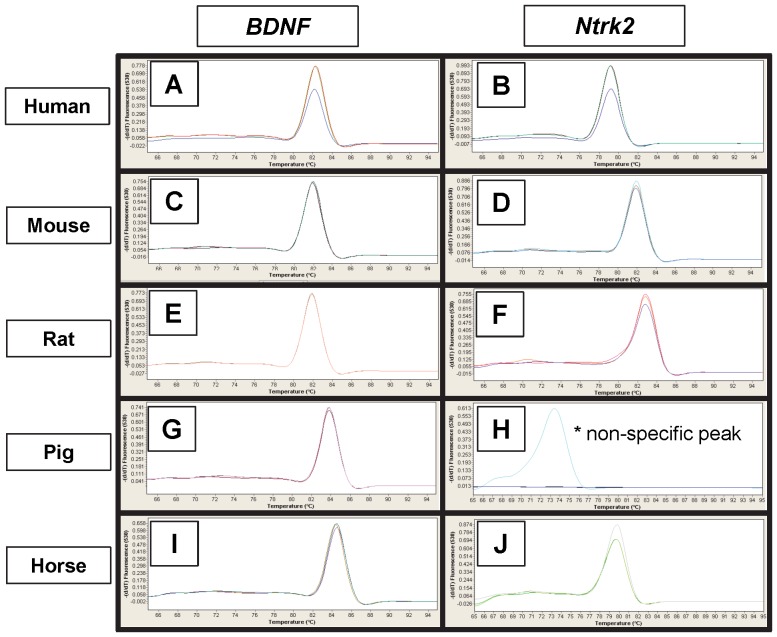
Isolation of Uterine BDNF and Ntrk2 Transcripts. Real-Time PCR melting peaks for uterine *BDNF* and *Ntrk2* in human (A, B), mouse (C, D), rat (E, F), pig (G, H), and horse (I, J). Both primer pairs isolated specific products which were verified by sequencing in all species except for a non-specific peak (*) obtained with the *Ntrk2* primers in pig uterus (H).

Primers designed against a 300 bp region of high homology within the *BDNF* and *Ntrk2* coding regions were used to isolate uterine transcripts by Real-Time PCR (Figure 2). Both primer pairs isolated specific products which were verified by sequencing in all species (human, mouse, rat, pig, and horse) except for a non-specific peak obtained with the *Ntrk2* primers in pig uterus. PCR product sequences were submitted to GenBank. Accession numbers are listed in Table 1.

### BDNF and Ntrk2 Antibody Specificity

#### Antibody Pre-absorption

**Figure 3:**
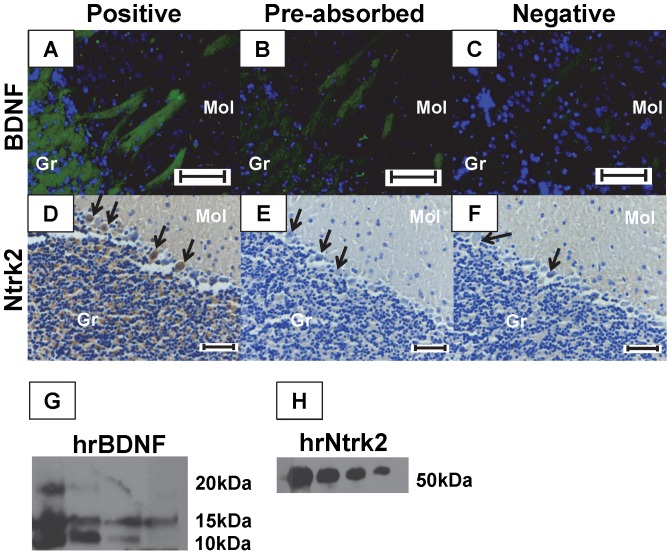
Assessing Antibody Specificity. Mouse brain sections were stained by immunohistochemistry with anti-BDNF (A) or Ntrk2 (D) antibodies as positive controls, or with antibody which had been pre-incubated with human recombinant BDNF (B) or Ntrk2 (E) protein, or with normal goat serum as a negative control (C, F). Decreased or absent staining was observed in pre-incubated sections as compared to positive controls (A vs. B; D vs. E). A 2X serial dilution of human recombinant BDNF (G) and truncated Ntrk2 (H) revealed bands of the appropriate sizes by Western Blot. Green: BDNF, brown: Ntrk2, blue: nucleus. Arrowheads: Purkinje cells, Gr: Granular layer, Mol: Molecular layer.

In order to confirm antibody specificity the antibodies used in this study were pre-absorbed using human recombinant proteins and used to stain mouse brain sections by immunohistochemistry. BDNF staining was minimized, and Ntrk2 staining was completely obliterated after antibody pre-absorption as compared to positive control sections (Figure 3A–F), indicating that the antibodies bound to their reported targets. Negative sections were included to show that minimal background staining was observed (Figure 3C,F).

#### Human Recombinant Protein Western Blot

The human recombinant BDNF and Ntrk2 which were used to pre-absorb the antibodies in 3.3.1 were examined by Western Blot in a 2X dilution. Specific bands of 10, 15, and 20 kDa were observed in the most concentrated dilution of BDNF (0.04 μg) (Figure 3G), and a band of approximately 50 kDa was observed in all dilutions of Ntrk2 (Figure 3H). The recombinant Ntrk2 protein was a truncated version of this receptor, and a band size of 50 was expected.

### BDNF and Ntrk2 Expression in the Uterus

#### Localization of BDNF and Ntrk2 by Immunohistochemistry

**Figure 4:**
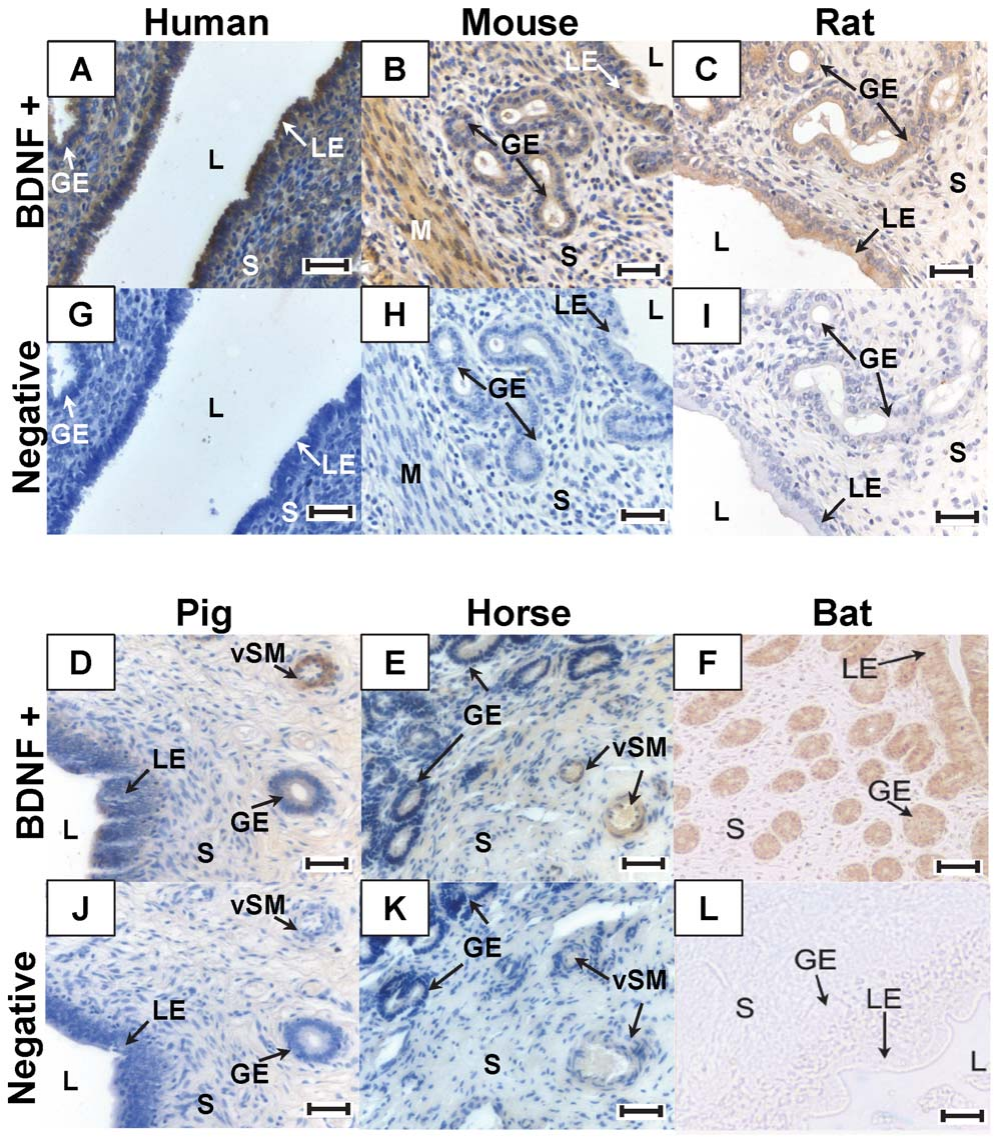
Immunohistochemical localization of BDNF in the Uterus. Uterine sections were stained for BDNF (A–F) using DAB as a chromogen (brown stain) or incubated with normal goat serum as a negative control (G–L). BDNF immunoreactivity was observed in human (A), mouse (B), rat (C), pig (D), horse (E), and bat (F) uterus. It localized to the luminal epithelium (LE), glandular epithelium (GE), smooth muscle of the myometrium (M) and vascular smooth muscle (vSM) in the mammals examined. Original image magnification was 200X. Scale bar represents 50 μm. L: lumen, S: stroma.

**Figure 5:**
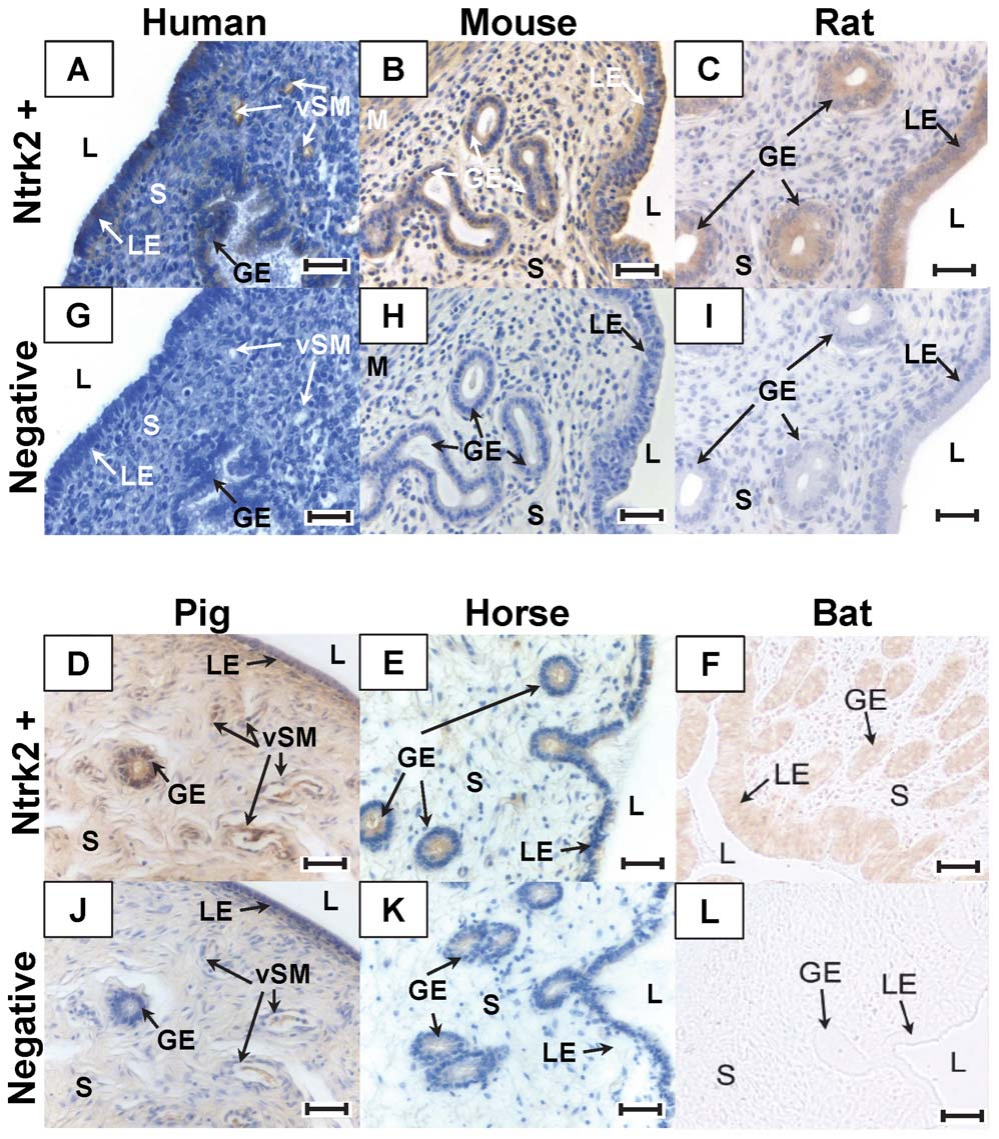
Immunohistochemical localization of Ntrk2 in the Uterus. Uterine sections were stained for Ntrk2 (A–F) using DAB as a chromogen (brown stain) or incubated with normal goat serum as a negative control (G–L). Ntrk2 immunoreactivity was observed in human (A), mouse (B), rat (C), pig (D), horse (E), and bat (F) uterus. It localized to the same areas as its ligand BDNF. Ntrk2 was observed in the luminal epithelium (LE), glandular epithelium (GE), smooth muscle of the myometrium (M) and vascular smooth muscle (vSM) in the mammals examined. Original image magnification was 200X. Scale bar represents 50 μm. L: lumen, S: stroma.

The uterine expression of BDNF (Figure 4) and Ntrk2 protein was assessed by immunohistochemistry (Figure 5). In all species examined (human, mouse, rat, pig, horse, and bat) BDNF immunoreactivity was detected in the luminal epithelium, glandular epithelium, myometrium, and vascular smooth muscle, particularly in pig and horse uterus. The uterine expression of Ntrk2 mirrored that of BDNF, being mainly localized in the luminal epithelium, glandular epithelium, and myometrium.

#### BDNF isolation in the Human Uterus by Western Blot

**Figure 6:**
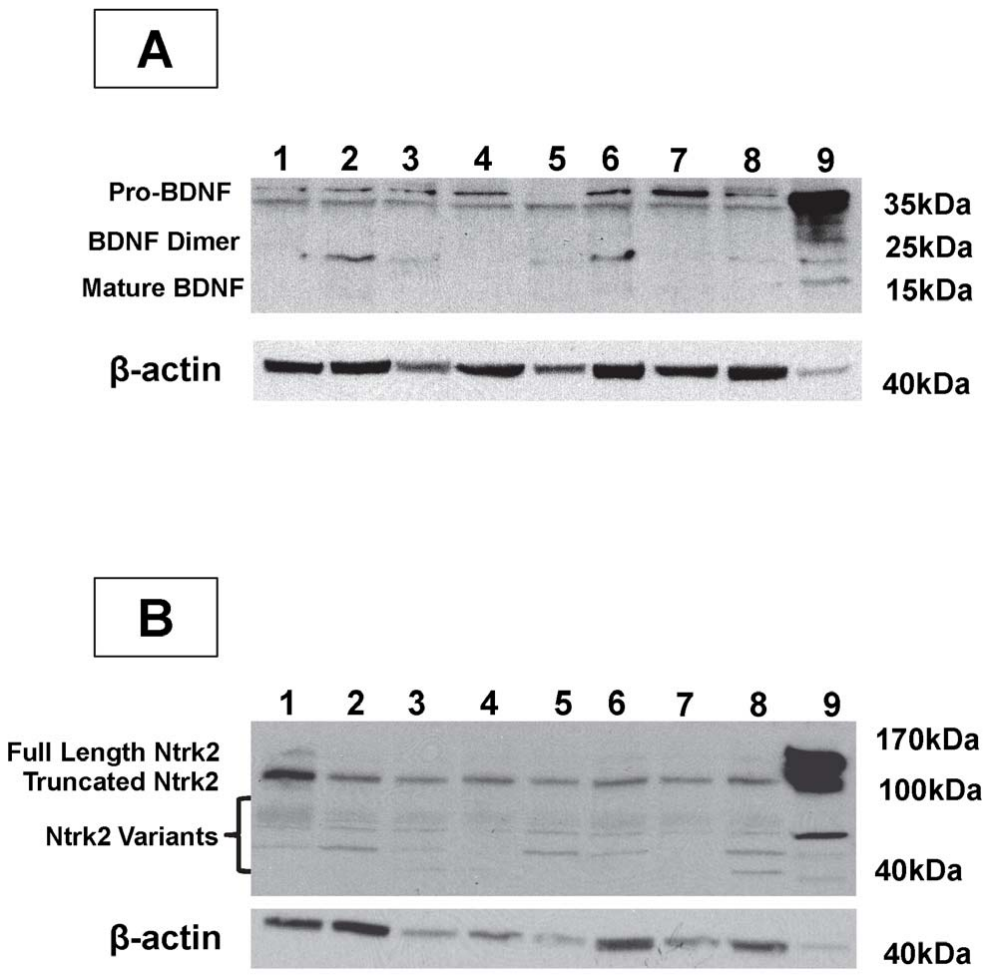
*BDNF and Ntrk2 Expression in the Human Uterus*. Uterine homogenates were collected from hysterectomy patients and probed for BDNF (A) and Ntrk2 (B) by Western Blot, using mouse brain as a positive control. Uterine samples were loaded in lanes 1–8, and mouse brain homogenate in lane 9.

Human endometrium from hysterectomy patients was probed for BDNF (Figure 6A) expression by Western Blot using mouse brain as a positive control. In all nine women, several bands were observed when the anti-BDNF antibody was used to probe the uterine homogenate. Faint 15 and 20 kDa bands were observed in some patients (Figure 6A) and in the mouse brain (Figure 6A: 9). A 25 kDa band was observed in the mouse brain, but not in the human uterus. A band of approximately 35 kDa was seen in all women, and in the mouse brain. However, in the uterine homogenates a doublet was found as compared to a single band in the mouse brain, and in patient 5. Blots were subsequently stripped and probed for beta-actin as a loading control.

#### Ntrk2 isolation in the Human Uterus by Western Blot

The same samples of human endometrium and mouse brain were probed for Ntrk2 (Figure 6B) expression by Western Blot. A single or double band of roughly 40 kDa were observed in some women (Figure 6B) and in mouse brain (Figure 6B: 9). A larger band of approximately 55 kDa, which was much more abundant in the mouse brain, was observed in all endometrial samples as a faint double band. A 100 kDa band which was heavily expressed in the mouse brain was observed in all uterine homogenates. Finally, a larger band of 120 kDa was seen in the positive control, and very faintly in a few of the human uteri. Blots were stripped and probed for beta-actin as a loading control.

## Discussion

Here, using complementary molecular techniques, we demonstrated the conservation of the coding region of BDNF and Ntrk2 across several mammalian species, the mRNA expression of both genes within the uterus, and the uterine localization of both proteins in two species that menstruate (humans and bats [34]), and four that do not (mice, rats, pigs, and horses). Additionally, we have shown that several protein isoforms of each gene were present in the human uterus, and that the antibodies employed in this study were specific to BDNF and Ntrk2 respectively. BDNF and Ntrk2 are part of the complex messenger system that is the neurotrophins, which regulate several physiological pathways, and thus we suggest are potentially important to uterine function.

Our results show that both BDNF and Ntrk2 are highly conserved across the mammalian species studied, with protein sequences having greater homology than mRNA sequences. This was not entirely unexpected, as in some cases multiple codons exist for a single amino acid, and thus a base-pair substitution in the mRNA sequence might not alter the protein. Over time, as each of the species studied evolved, silent mutations in the genes likely arose. During evolution, Gotz et al., suggest that BDNF was more highly conserved than NGF across vertebrates [35]. In our study the PCR primer pairs designed to isolate *BDNF* and *Ntrk2* were capable of doing so in the uterus of all animals (except for *Ntrk2* in the porcine uterus), and both antibodies employed in this study demonstrated specific uterine immunoreactivity for BDNF and Ntrk2 in each of the six mammals examined, supporting high sequence homology amongst orthologs over evolution.

Antibody specificity in the current study was ascertained in two ways. Firstly, by ensuring bands of the appropriate size were seen when Western blot was performed with human recombinant BDNF and Ntrk2. Secondly, mouse brain sections (positive control tissue) were stained for BDNF and Ntrk2 with primary antibodies which had been pre-absorbed with BDNF and Ntrk2, respectively. In sections incubated with pre-absorbed BDNF primary antibodies the staining was less intense than the positive control, which had been stained with anti-BDNF, but more intense than the negative control. Ideally pre-absorption obliterates all staining as the antibody should be completely bound by the excess protein. In the case of pre-absorbed BDNF, some of the BDNF bound to the anti-BDNF antibody may have bound to endogenous Ntrk2 receptors, and thus given a faint signal when the secondary antibody was applied. Ntrk2 staining in the mouse brain was obliterated by pre-absorption. The results of the antibody specificity tests indicated that the antibodies used for immunohistochemistry and Western blot were specific and capable of detecting BDNF and Ntrk2 within the mammalian uterus.

While there are a few studies demonstrating the independent expression of BDNF and Ntrk2 in the uterus, the results of the present study are the first to show that both ligand and receptor are co-expressed, and co-localized in the uterus of several mammalian species. Our results show BDNF and Ntrk2 expression in the glandular epithelium, luminal epithelium, vascular smooth muscle, and myometrium of the human, mouse, rat, pig, and bat uterus. A similar pattern of expression was also observed in the uterus of the pregnant mare. This is the first comprehensive and cross-species comparison of BDNF and Ntrk2 mRNA, and protein in the mammalian uterus. Even though BDNF expression has been seen in uterine pathologies [36], [37], BDNF and Ntrk2 expression in the non-pregnant, healthy uterus has been equivocal. Although there are sparse reports of BDNF in the mouse [13], [25], rat [18], and human [26], [27] uterus, and Ntrk2 in the human uterus [28], [29], others have not been able to localize the Ntrk receptor family in the murine [13] nor human uterus [15]. However, the latter study [15] published in 1996, may not have been able to detect Ntrk2 owing to limitations in the sensitivity of PCR techniques then available. Additionally, the co-localization of BDNF and Ntrk2 demonstrated in this study contrasts the results of Lommatzsch et al. (1999) [13], where *BDNF* mRNA was only observed in the uterine epithelium and stroma, not myometrium, and Ntrk2 immunoreactivity was not observed at all. Again, this may have been due to methodological limitations. The probe designed for *in situ* hybridization may not have detected all forms of BDNF (pre-, pro-, etc.), and if that particular form was present in the myometrium it would have falsely appeared negative. Also, Ntrk2 appears to exist in low abundance in the uterus; the exposure length to obtain a positive Western blot band is one hour, after loading 60 μg of protein homogenate. Perhaps the antibody used in the previous report was not as sensitive as the antibody employed in this study.

Neurotrophin signalling and regulation is complicated for several reasons: each receptor can bind more than one ligand with varying affinity, multiple splice and transcript variants of ligands and receptors exist, several post-translational modifications may be present, ligands are first translated as pro-proteins which bind receptors, and ligands can exist as monomers or dimers [1]. Thus, the expression of BDNF and Ntrk2 were demonstrated by Western blot in the human endometrium to gain insight into which isoform predominates. A doublet band of roughly 35 kDa was found to be the most widely expressed form of BDNF in the uterus. These bands are likely pro-BDNF which has previously been reported to have a similar mass [38], [39]. Smaller bands of approximately 15 kDa likely represent the mature form of BDNF, and are less abundant than the larger bands. It has been suggested that pro-BDNF and mature BDNF have opposing functions. Specifically, pro-BDNF inhibits nerve growth and BDNF promotes and sustains it [40], [41]. As for Ntrk2, variability was seen between patients for the bands lower than 100 kDa, but a band at approximately 100 kDa was consistent amongst them all. This band likely represents a truncated version, of which there are two at 95 kDa, of the 140 kDa receptor [42]–[46].

We speculate that the abundant BDNF and Ntrk2 isoforms found in the human uterus may serve to inhibit the classical BDNF-Ntrk2 pathways, and also prevent nerve growth into a tissue that is degraded and shed in a cyclical manner. However, the degree to which nerves innervate the endometrial layer of the uterus under physiological and pathological conditions remains under debate [47]–[50]. In support of our hypothesis, expression of the truncated Ntrk2 was capable of inhibiting sensory nerve innervation of the mammary gland in response to mature BDNF [51]. BDNF and Ntrk2 have also previously been shown to activate the adhesion [5], [52]–[55], angiogenesis [56], [57], apoptosis [5], [53], [58]–[60], and proliferation [59], [61] pathways, mainly in the brain and nervous system. Each of these pathways is also of paramount importance in the reproductive processes of the female mammal. However, little is known about the role of BDNF and Ntrk2 in reproductive physiology. While the literature supporting BDNF expression, particularly in the brain and serum, during pregnancy is growing [62]–[65], its specific function is still unclear. One group has reported that paracrine BDNF/Ntrk2 signalling induced cytotrophoblast differentiation, proliferation, and survival in an *in vitro* model [25], [30], while another showed that BDNF inhibited neurite outgrowth in a superior cervical ganglion/myometrium explant co-culture [18]. While the role of BDNF/Ntrk2 in reproductive physiology remains a mystery we suggest that this signaling pathway is potentially important in normal uterine physiology and pathology.

Herein we have given a complete and comprehensive overview of BDNF and Ntrk2 in the mammalian uterus. Firstly, gene conservation was demonstrated for both BDNF and Ntrk2 across species. Secondly, transcripts for both *BDNF* and *Ntrk2* were isolated in the uterus of several mammals. Thirdly, the antibodies were confirmed to be specific for the proteins of interest. Fourthly, protein translation and localization was demonstrated by immunohistochemistry in menstruating and non-menstruating species, and finally the prominent BDNF and Ntrk2 isoforms were identified in the human endometrium. As several of the major pathways central to reproductive biology have been reported to be induced by BDNF-Ntrk2 binding, we suggest that the function of this ligand-receptor pair within the mammalian uterus merits further attention.
